# Awareness and Attitude Toward Epidural Analgesia During Labor Among Pregnant Women in Taif City: A Hospital-Based Study

**DOI:** 10.7759/cureus.49367

**Published:** 2023-11-24

**Authors:** Alaa M Abdelhafeez, Fahad K Alomari, Hassan M Al Ghashmari, Ahmed Newera, Hussain O Alshehri, Fahad M Alzulfi, Khaled A Khreisat, Awad A Osman, Mardi A Osman

**Affiliations:** 1 Department of Anesthesia, Prince Sultan Military Hospital, Taif, SAU; 2 Department of Anesthesia and Critical Care, Faculty of Medicine, Omdurman Islamic University, Khartoum, SDN; 3 Specialization of Anesthesia, The College of Anaesthesiologist of Ireland (FCAI), Duplin, IRL; 4 Specialization of Anesthesia and Intensive Care, Jordanian Board, Amman, JOR; 5 Specialization of Anesthesia and Intensive Care, Arab Board, Amman, JOR; 6 Department of Family Medicine, Prince Sultan Military Hospital, Taif, SAU; 7 Specialization of Family Medicine, Saudi Board, Riyadh, SAU; 8 Specialization of Family Medicine, Arab Board, Riyadh, SAU; 9 Specialization of Health Sciences, King Abdulaziz University, Jeddah, SAU; 10 Department of Continuous Quality Improvement and Patient Safety, Prince Sultan Military Hospital, Taif, SAU; 11 Specialization of Social and Preventive Medicine, University of Malaya, Kuala Lumpur, MYS; 12 Department of Anaesthesia, Taif and Alhada Military Hospital, Taif, SAU; 13 Specialization of Anaesthesia, King Saud University Board, Riyadh, SAU; 14 Department of Anaesthesia, Prince Sultan Military Hospital, Taif, SAU; 15 Department of Obstetrics and Gynaecology, Prince Sultan Military Hospital, Taif, SAU; 16 Specialization of Obstetrics and Gynaecology, The Royal College of Obstetricians and Gynaecologists, London, GBR

**Keywords:** taif city, labor pain, epidural analgesia, pregnant women, attitude, awareness

## Abstract

Objectives

This study aimed to investigate the awareness and attitudes towards epidural analgesia (EA) among pregnant women in Taif City, Saudi Arabia. The rationale was to identify potential barriers to the acceptance and use of EA, which is an effective pain management option during labor.

Methods

We conducted a cross-sectional survey at a single healthcare center in Taif City. The participants, pregnant women visiting the center, were recruited using a convenience sampling method. Data collection was facilitated by a questionnaire distributed through a quick response (QR) code. The questionnaire assessed demographic information, awareness levels, previous exposure to EA, and personal attitudes toward its use during labor. Data analysis focused on quantifying the levels of awareness and identifying patterns in attitudes.

Results

The results revealed a low level of awareness about EA among the participants, with a significant proportion having never been exposed to it before the survey. Attitudes towards EA were varied, with some expressing openness to its use and others displaying apprehension or resistance, which appeared to be influenced by cultural perceptions and a lack of information.

Conclusions

The study highlighted a substantial lack of awareness and varied attitudes towards EA among pregnant women in Taif City. Educational interventions are necessary to increase awareness and address cultural misconceptions. The study’s limited scope and potential sample bias suggest the need for broader culturally tailored research to inform strategies for improving the acceptance and utilization of labor analgesia.

## Introduction

Labor pain is among the most intense pains encountered by humans. Yet its recollection fades over time. Unlike other types of pain, labor pain is a profoundly personal and subjective experience that is challenging but also emotional and significant. Cognitive, social, and environmental factors are the pivotal elements shaping the perception of labor pain. Notably, even with the intense nature of labor pain, the administration of labor analgesia during childbirth is not a standard practice. Multiple studies have underestimated the role of labor analgesia in contributing to childbirth satisfaction, as it is perceived to play a minor part. However, if a woman maintains the belief that her pain is both purposeful (i.e., her body’s effort to deliver her baby) and productive (i.e., progressing her toward a desired outcome), and if she perceives the birthing environment as safe and supportive, she may view the pain as a non-threatening and transformative event. This perception of labor pain as purposeful and productive can enhance a woman’s labor pain experience and diminish the demand for pain interventions. Nonetheless, there are concerns about the safety of labor analgesia, particularly with regard to its effects on labor progress and outcomes. Epidural analgesia (EA), a common method, is thought to prolong the second stage of labor and increase instrumental vaginal delivery rates [1-3].

Conversely, many studies advocate for labor analgesia, especially neuraxial labor analgesia, which includes epidurals and combined epidural-spinal techniques, due to its effectiveness in pain relief [1]. EA, which administers a local anesthetic near pain-transmitting nerves, is generally well-received by healthy young women due to its effectiveness and low complication rate. Furthermore, it has been associated with a decreased incidence of postpartum depression. EA also facilitates regional anesthesia for obstetric procedures such as forceps deliveries and cesarean sections (C/S), benefiting women at risk for these interventions, like those with obesity [1,4-6]. Evidence suggests that effective labor analgesia does not raise C/S rates [3]. Akadri and Odelola promoted offering labor analgesia to those desiring it, thereby enhancing the childbirth experience [7]. Similarly, Aziato et al. emphasized the need for healthcare professionals to manage labor pain effectively within a sociocultural context [8]. Despite ongoing debates, it is widely accepted that labor analgesia should be customized to each woman’s individual needs, preferences, and circumstances, including anticipated labor duration, the condition of the infant, and any procedures to induce or augment labor [6-8].

Awareness among pregnant women is crucial in shaping their attitudes toward labor analgesia in general and EA in particular, highlighting the importance of education for expecting mothers [8]. This study was undertaken at Prince Sultan Military Hospital in Taif City, located in the western region of Saudi Arabia, to assess pregnant women’s awareness of EA and to identify factors influencing their attitudes towards its use, comparing these with other regions within Saudi Arabia.

## Materials and methods

Study design

This cross-sectional, prospective, single-center, hospital-based study was conducted with pregnant women at the obstetrics outpatient clinic of Prince Sultan Military Hospital in Taif City, western Saudi Arabia. The Taif and Alhada Military Hospital’s Research Ethical Committee approved the study (Approval No. REC.2023-704).

Sample population

We estimated a sample size of 478 pregnant women using the Raosoft Sample Size Calculator (Raosoft, Inc., Seattle, WA) for the six-month data collection period, aiming for a confidence level above 97% and a margin of error below 5%. Participation was contingent upon informed consent, granted after participants were briefed on the study’s aims and methods. We included pregnant women attending routine antenatal care who agreed to participate and excluded those unable to scan the questionnaire’s quick response (QR) code with their mobile phones.

Data collection

Data were gathered through a self-administered questionnaire, distributed in Arabic, and accessible via a QR code scanned by the participant’s mobile phone. Participants were allowed only one attempt to complete the questionnaire to avoid duplicate responses. Before the main study, the questionnaire was pretested and validated on a pilot group of 50 pregnant women, who were subsequently excluded from the primary analysis. The data collection occurred from February 1, 2023, to July 31, 2023.

The questionnaire comprised three sections, starting with a consent statement. The first section collected demographic information, including age, nationality, education level, employment status, and monthly income. The second section focused on medical and obstetric history, querying participants about medical comorbidities such as diabetes, gestational diabetes (GD), hypertension (HTN), preeclampsia, cardiac disease, asthma, hypothyroidism, the number of prior births, and previous C/S. The third section assessed specific knowledge about EA, including history of EA use in previous pregnancies, willingness to consider EA in the current pregnancy or recommend it to others, understanding of EA’s mechanisms (e.g., whether it affects uterine contractions or the ability to push, leading to C/S, or if lower limb paralysis is a complication), and preferred methods for acquiring further information about EA (e.g., no further education, videos, brochures, consultations with an obstetrician during antenatal visits, or teaching sessions by an anesthetist).

## Results

Statistical analysis

We conducted statistical analyses using IBM SPSS Statistics for Windows, Version 28.0 (Armonk, NY: IBM Corp.). We summarized categorical variables by frequency and percentage and analyzed them with the Chi-square test. We applied univariate and multivariable logistic regression models to examine the factors influencing knowledge about EA. We deemed a p-value of less than 0.05 as indicative of statistical significance.

Results

Our study comprised 478 pregnant women from the obstetrics outpatient clinic at Prince Sultan Military Hospital in Taif City, Saudi Arabia. Most of these women were aged between 21 and 40 (86.1%), with 97.7% being Saudi nationals. Over half held a university degree (54.3%), while a substantial proportion were unemployed (87.8%). The prevalent monthly income bracket was between 5,000 and 10,000 Saudi Riyals (SAR), accounting for 79.1% of the participants. A significant portion (71.8%) had no underlying medical conditions. However, 14.0% had diabetes or GD, 6.9% were diagnosed with hypothyroidism, 4.0% had HTN or preeclampsia, 3.6% had asthma, and a minority of two participants (0.4%) had cardiac diseases. Concerning childbirth history, 39.2% had given birth more than twice, 38.1% one or two times, and 22.7% had not given birth previously. Additionally, 29.5% had previously undergone a C/S (Table 1).

**Table 1:** Participant demographic data (n= 478) C/S, cesarean section; GD, gestational diabetes; HTN, hypertension; SAR, Saudi Riyals.

Demographic data		n	%
Age (years)	≤20	8	1.7
	21–30	223	46.8
	31–40	187	39.3
	>40	58	12.2
Nationality	Saudi	466	97.7
	Non-Saudi	11	2.3
Education level	Primary school	73	15.3
	Secondary school	145	30.4
	University education or more	259	54.3
Employment status	Unemployed	418	87.8
	Employed in a medical profession	31	6.5
	Employed in a non-medical profession	27	5.7
Monthly income (SAR)	5,000 to 10,000	280	79.1
	>10,000 to 15,000	50	14.1
	>15,000	24	6.8
Background medical condition	None	343	71.8
	Diabetes or GD	67	14.0
	HTN or pre-eclampsia	19	4.0
	Cardiac disease	2	0.4
	Asthma	17	3.6
	Hypothyroidism	33	6.9
Parity status	0	108	22.7
	1–2	181	38.1
	>2	186	39.2
History of C/S	No	335	70.5
	Yes	140	29.5

Only 25 participants (5.3%) had previous experience with EA. Approximately half of the participants (53.3%) were open to considering EA during labor. Regarding beliefs about EA, 21.6% believed that EA could reduce or halt uterine contractions, 7.0% thought it could lead to serious complications like paraplegia, and 5.1% believed that it could impede the mother’s ability to push, potentially necessitating a C/S. We defined adequate knowledge of EA as correctly answering at least two out of three questions on the subject. Only 6.7% (32 participants) demonstrated good knowledge of EA. When asked about preferred methods of learning about EA in the future, only 12.7% felt no need for further education (Table 2).

**Table 2:** Responses to questions assessing specific knowledge of EA C/S, cesarean section; EA, epidural analgesia.

Question	Answer	n	%
Have you ever had EA before?	Yes	25	5.3
	No	450	94.7
Are you considering receiving EA during labor?	Yes	252	53.3
	No	60	12.7
	Don't know	161	34.0
	Yes	102	21.6
Does EA minimize or stop uterine contractions?	No	41	8.7
	Don't know	329	69.7
Does EA prevent a mother from pushing and lead to C/S?	Yes	24	5.1
	No	108	22.8
	Don't know	341	72.1
Does EA cause serious complications like paraplegia?	Yes	33	7.0
	No	119	25.2
	Don't know	320	67.8
Knowledge regarding EA (based on the previous three questions)	Good	32	6.7
	Poor	446	93.3
Would you like future education about EA?	Yes	419	87.7
	No	59	12.7

**Figure 1:**
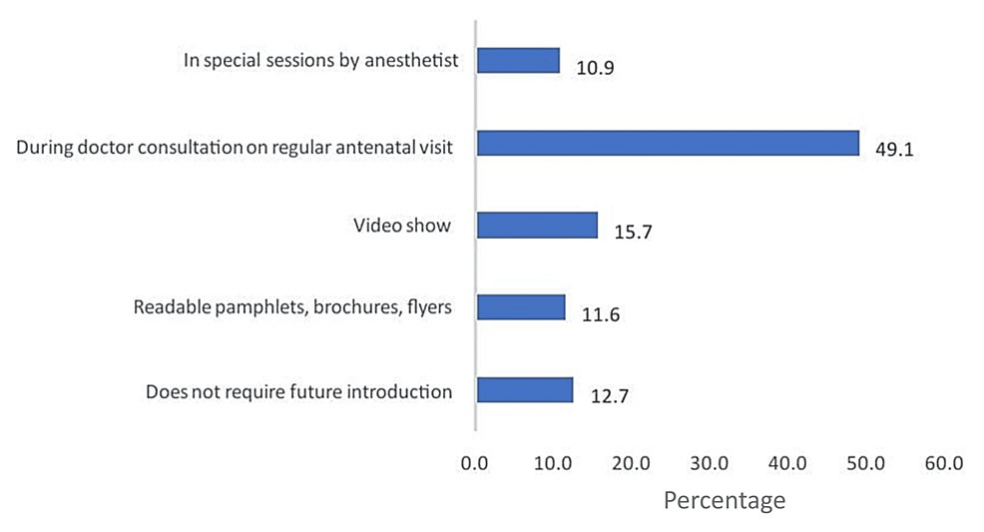
Preferred method of future education about epidural analgesia

The most favored method of education was through regular consultations with obstetricians during antenatal visits (49.1%), followed by video presentations (15.7%), written materials such as pamphlets, brochures, and flyers (11.6%), and special sessions led by an anesthetist (10.9%; Figure 1).

**Figure 2:**
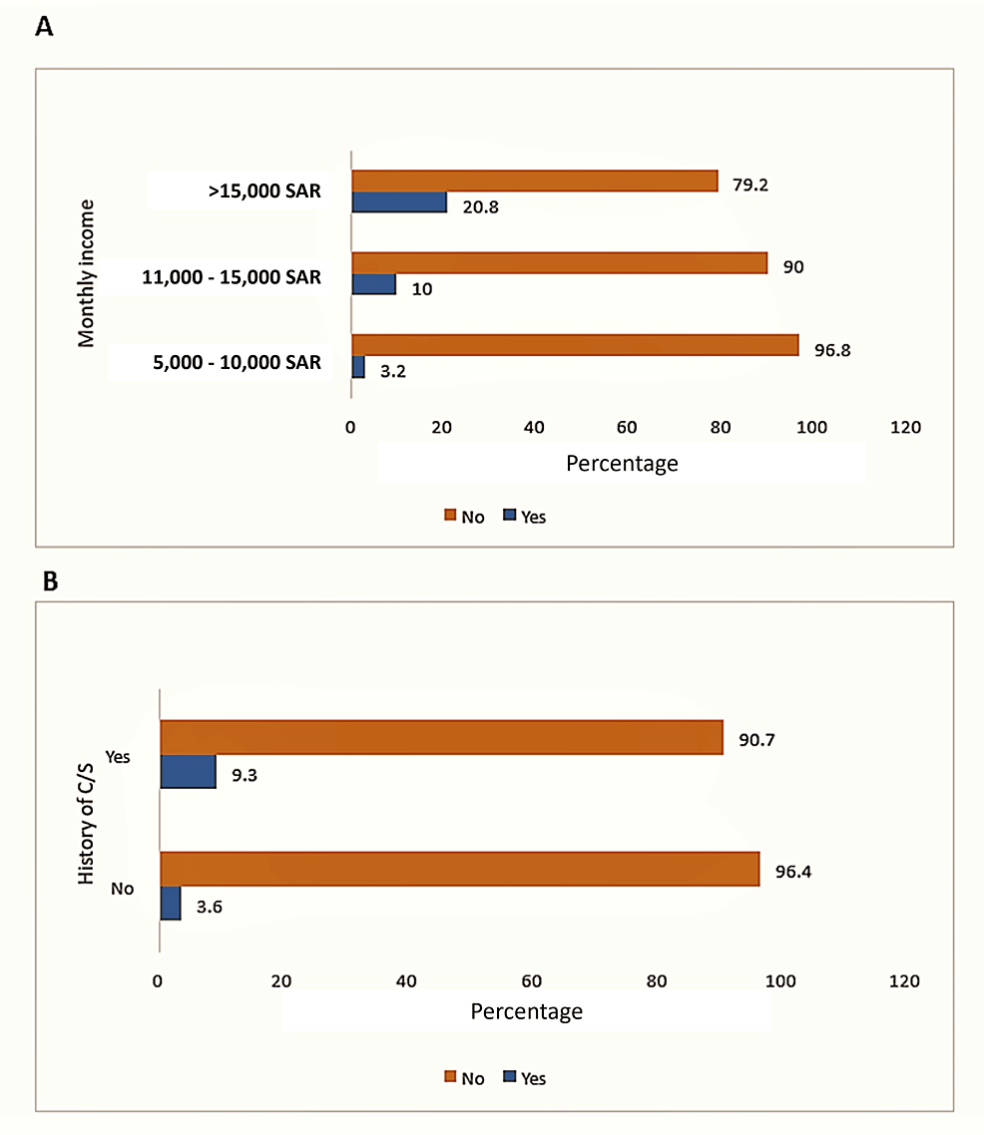
Previous trial of epidural analgesia across different monthly income groups (A) and with or without a history of C/S (B) C/S, cesarian section; SAR, Saudi Riyals.

We further analyzed participant characteristics based on prior receipt of the EA. Those who had received EA were significantly more likely to have a higher monthly income (20.8% in the >15,000 SAR category, 10.0% in the >10,000 to 15,000 SAR category, and 3.2% in the 5,000 to 10,000 SAR category; p = 0.002). The prevalence of EA use was also higher among participants who had a previous C/S than those who had not (9.3% vs. 3.6%, respectively; p = 0.012; Table 3, Figure 2).

**Table 3:** Association of participant demographic data with previous epidural analgesia C/S, cesarean section; GD, gestational diabetes; HTN, hypertension; SAR, Saudi Riyals. *Significant at p<0.05 using chi-square test and exact test.

Demographic data		Previous epidural analgesia		P-value
		Yes, n (%)	No, n (%)	
Age (years)	≤30	9 (3.9)	221 (96.1)	0.428
	31–40	12 (6.5)	173 (93.5)	
	>40	4 (6.9)	54 (93.1)	
Nationality	Saudi	24 (5.2)	439 (94.8)	>0.999
	Non-Saudi	1 (9.1)	10 (90.9)	
Education level	Primary school	3 (4.1)	70 (90.9)	0.815
	Secondary school	7 (4.9)	137 (95.1)	
	University education or more	15 (5.8)	242 (94.2)	
Employment status	Unemployed	22 (5.3)	393 (94.7)	>0.999
	Employed in a medical profession	2 (6.5)	29 (93.5)	
	Employed in a non-medical profession	1 (3.7)	26 (96.3)	
Monthly income (SAR)	5,000–10,000	9 (3.2)	269 (96.8)	0.002*
	>10,000 to 15,000	5 (10.0)	45 (90.0)	
	>15,000	5 (20.8)	19 (79.2)	
Background medical condition	No	5 (3.8)	128 (96.2)	0.36
	Yes	20 (5.8)	322 (94.2)	
Diabetes or GD	No	21 (5.1)	387 (94.9)	>0.999
	Yes	4 (6.0)	63 (94.0)	
HTN or pre-eclampsia	No	24 (5.3)	432 (94.7)	>0.999
	Yes	1 (5.3)	18 (94.7)	
Cardiac disease	No	24 (5.1)	449 (94.9)	0.103
	Yes	1 (50.0)	1 (50.0)	
Asthma	No	25 (5.5)	449 (94.9)	0.616
	Yes	0 (0.0)	17 (100.0)	
Hypothyroidism	No	24 (5.4)	418 (94.6)	0.715
	Yes	1 (3.0)	32 (97.0)	
Parity status	0	2 (1.9)	105 (98.1)	0.194
	1-2	11 (6.1)	170 (93.9)	
	>2	12 (6.5)	172 (93.5)	
History of C/S	No	12 (3.6)	321 (96.4)	0.012*
	Yes	13 (9.3)	127 (90.7)	

When considering the inclination towards EA during labor, younger participants were more likely to consider it (61.1% under 30, 51.1% aged 31 to 40, and 29.7% over 40; p < 0.001). The likelihood of considering EA was also significantly associated with the level of education; those with a university degree were more inclined (63.7%) than those with secondary (46.2%) or primary education (31.5%; p < 0.001). Furthermore, participants with HTN or preeclampsia were less likely to consider EA (36.8%) than those without these conditions (54.0%; p = 0.037). Parity also showed a significant association; women who had given birth once or twice were more likely to consider EA (61.9%) compared to those who had never given birth (57.1%) and those who had given birth more than twice (42.7%; p < 0.001; Table 4).

**Table 4:** Association of participant demographic data with consideration of epidural analgesia during labor C/S, cesarean section; GD, gestational diabetes; HTN, hypertension; SAR: Saudi Riyals. *Significant at p<0.05 using the chi-square test and the exact test.

Demographic data		Responses to “Are you considering epidural analgesia during labor?”			P-Value
		Yes, n (%)	No, n (%)	I don’t know, n (%)	
Age (years)	≤30	140 (6.1)	18 (7.9)	71 (31.0)	<0.001*
	31–40	94 (51.1)	30 (16.3)	60 (32.6)	
	>40	17 (29.3)	12 (20.7)	29 (50.0)	
Nationality	Saudi	245 (53.1)	58 (12.6)	158 (34.3)	0.842
	Non-Saudi	6 (54.5)	2 (18.2)	3 (27.3)	
Education level	Primary school	23 (31.5)	20 (27.4)	30 (41.1)	<0.001*
	Secondary school	66 (46.2)	19 (13.3)	58 (40.6)	
	University education or more	163 (63.7)	21 (8.2)	72 (28.1)	
Employment status	Unemployed	219 (52.9)	51 (12.3)	144 (34.8)	0.623
	Employed in a medical profession	16 (53.3)	6 (20.0)	8 (26.7)	
	Employed in a non-medical profession	16 (59.3)	2 (7.4)	9 (33.3)	
Monthly income (SAR)	5,000–10,000	144 (51.4)	37 (113.2)	99 (35.4)	0.320
	>10,000 to 15,000	24 (50.0)	7 (14.6)	17 (35.4)	
	>15,000	14 (58.3)	6 (25.0)	4 (16.7)	
Background medical condition	No	64 (48.9)	19 (14.5)	48 (36.6)	0.473
	Yes	188 (55.0)	41 (12.0)	113 (33.0)	
Diabetes or GD	No	220 (54.1)	49 (12.0)	138 (33.9)	0.521
	Yes	32 (48.5)	11 (16.7)	23 (34.8)	
HTN or pre-eclampsia	No	245 (54.0)	54 (11.9)	155 (34.1)	0.037*
	Yes	7 (36.8)	6 (31.6)	6 (31.6)	
Cardiac disease	No	251 (53.3)	60 (12.7)	160 (34.0)	>0.999
	Yes	1 (50.0)	0 (0.0)	1 (50.0)	
Asthma	No	244 (53.5)	58 (12.7)	154 (33.8)	0.817
	Yes	8 (47.1)	2 (11.8)	7 (41.2)	
Hypothyroidism	No	239 (54.3)	56 (12.7)	145 (33.0)	0.177
	Yes	13 (39.4)	4 (12.1)	16 (48.5)	
Parity status	0	60 (57.1)	6 (5.7)	39 (37.1)	<0.001*
	1–2	112 (61.9)	14 (7.7)	55 (30.4)	
	>2	79 (42.7)	40 (21.6)	66 (35.7)	
History of C/S	No	178 (53.6)	38 (11.4)	116 (34.9)	0.506
	Yes	74 (53.2)	21 (15.1)	44 (31.7)	

Using univariate logistic regression, we found that age, nationality, and employment status were significantly associated with knowledge about EA. Participants aged 31 to 40 were more likely to have good knowledge than those 30 and younger (odds ratio [OR] = 2.26, 95% confidence interval [CI] for OR: 1.05, 4.88; p = 0.038). Non-Saudi participants were more knowledgeable than Saudis (OR = 5.65, 95% CI for OR: 1.42, 22.44; p = 0.014). Employed participants, particularly those in medical professions, showed higher knowledge levels than unemployed participants (OR = 6.26, 95% CI for OR: 2.25, 15.58; p < 0.001; Table 5).

**Table 5:** Univariate logistic regression analysis of factors affecting epidural analgesia knowledge CI, confidence interval; OR, odds ratio; C/S, cesarean section; GD, gestational diabetes mellitus; HTN, hypertension; NA, not applicable; SAR, Saudi Riyals. *Significant at p<0.05 using chi-square test and exact test.

Factors		Unadjusted OR	P-value	95% CI	OR
Age (year)	≤30	Ref.	NA	NA	NA
	31–40	2.26	0.038*	1.05	4.88
	>40	0.71	0.667	0.15	3.32
Nationality	Saudi	Ref.	NA	NA	NA
	Non-Saudi	5.65	0.014*	1.42	22.44
Education level	Primary school	Ref.	NA	NA	NA
	Secondary school	1.53	0.607	0.30	7.79
	University education or more	3.63	0.085	0.84	15.72
Employment status	Unemployed	Ref.	NA	NA	NA
	Employed in a medical profession	6.26	<0.001*	2.52	15.58
	Employed in a nonmedical profession	1.44	0.634	0.32	6.47
Monthly income (SAR)	5,000–10,000	Ref.	NA	NA	NA
	>10,000 to 15,000	1.19	0.756	0.39	3.67
	>15,000	1.96	0.308	0.54	7.17
Background medical condition	None	1.44	0.410	0.61	3.41
	Diabetes or GD	0.87	0.798	0.29	2.56
	HTN or preeclampsia	0.77	0.799	0.10	5.94
	Cardiac disease	0.00	0.999	0.00	0.00
	Asthma	0.87	0.892	0.11	6.75
	Hypothyroidism	0.89	0.880	0.20	3.91
Parity	0	Ref.	NA	NA	NA
	1–2	0.84	0.738	0.31	2.29
	>2	1.27	0.620	0.50	3.21
History of C/S	No	Ref.	NA	NA	NA
	Yes	0.53	0.175	0.21	1.32

Multivariable logistic regression indicated that employment status, particularly in the medical field, was a significant predictor of possessing good knowledge about EA, even after adjusting for other factors (OR = 3.16, 95% CI for OR: 1.03, 9.69; p = 0.045; Table 6).

**Table 6:** Multivariable logistic regression analysis of factors affecting epidural analgesia knowledge CI, confidence interval; OR, odds ratio; C/S, cesarean section; NA, not applicable. *Significant at p<0.05 using using chi-square test and exact test.

Factors		Adjusted OR	P-value	95% CI	OR
Age (years)	≤30	Ref.	NA	NA	NA
	31−40	2.16	0.068	0.95	4.96
	>40	1.03	0.973	0.20	5.25
Nationality	Saudi	Ref.	NA	NA	NA
	Non-Saudi	1.37	0.707	0.27	6.95
Education level	Primary school	Ref.	NA	NA	NA
	Secondary school	1.56	0.596	0.30	8.05
	University education or more	2.63	0.219	0.56	12.31
Employment status	Unemployed	Ref.	NA	NA	NA
	Employed in a medical profession	3.16	0.045*	1.03	9.69
	Employed in a non-medical profession	1.08	0.927	0.22	5.26
History of C/S	No	Ref.	NA	NA	NA
	Yes	0.53	0.188	0.21	1.37

## Discussion

Saudi Arabia is a vast country with myriad geographic and cultural characteristics [9]. This hospital-based study conducted in Taif City, located in the western region of Saudi Arabia, aimed to explore pregnant women’s awareness and attitudes toward EA during labor in comparison with other regions of the Kingdom. Our sample’s demographics align with those from similar studies across the country [10-18]. Interestingly, only employment status - specifically being employed in the medical field - was a significant predictor of awareness about EA, diverging from another study in the same region that implicated age, education, history of C/S, and previous EA experiences as influential factors [11]. Using multivariable logistic regression, our analysis determined that employment status was the primary determinant. Awareness of EA among the women in our study was low at 6.7%, notably less than percentages reported from other Saudi regions, where the figures ranged from 16.2% to 85.6% [10-18]. This disparity could be due to Taif’s predominantly rural population, where traditional views may prioritize natural over medical approaches, impacting knowledge acquisition about EA. A clear link between knowledge of EA and its utilization has been established [10,11,13,16-18]. Moreover, only 5.3% of our study participants had previously been exposed to EA, the lowest rate compared to other Saudi regions where exposure rates ranged from 20.1% to 38.6% [10-13,15,16,18]. Moreover, only 5.3% of our study participants had previously been exposed to EA, the lowest rate compared to other Saudi regions where exposure rates ranged from 20.1% to 38.6% [10-13,15,16,18]. This may reflect the limited availability of EA in local healthcare centers and underscore the need for enhanced awareness. We noted a significant correlation between a higher monthly income (over 15,000 SAR) and a previous receipt of EA. However, unlike findings from a study in Jazan that showed a correlation between EA exposure and women aged 30 to 40 living in urban areas with higher educational levels [12], we did not find a significant correlation with other demographic variables. This suggests that economic factors may influence the accessibility of EA.

Contrary to existing evidence suggesting that EA does not increase the C/S delivery rate [3], our study revealed a notable association between the C/S rate and prior exposure to EA. This unexpected discovery highlights the need for further research to confirm these findings and to re-evaluate our practices in Taif City.

The C/S rate in our study was 29.5%, situated between rates found in other regional studies [11,15]. It has been suggested that increasing awareness of labor analgesia, specifically EA, might reduce the rate of elective C/S deliveries, which is fueled by the fear of labor pain [19]. Regarding preferences for EA, 53.3% of participants were considering it for their current pregnancy. This desirability rate for EA indicates some acceptance of the method in Taif, albeit lower than in some regions but higher than in others [10,12,13]. Younger women, those with fewer children, and university graduates showed a higher likelihood of choosing EA, contrasting with findings from Al Khobar [10].

Medical conditions in our participants did not seem to influence awareness or previous use of EA. However, women with HTN or preeclampsia were less inclined to desire EA, contrary to our initial assumption that medical advice would increase awareness in such cases. The implications of the EA for patients with specific medical conditions warrant further investigation.

Educating pregnant women is pivotal in navigating labor analgesia choices. Inclusion of early education on labor analgesia in antenatal care, delivered by anesthetists and obstetricians, is recommended [10-12]. Our findings revealed that the preferred source of education about EA was obstetricians, which highlights the importance of direct physician-patient interaction. Following this study, we initiated integrated lectures by anesthetists and obstetricians as part of the antenatal care visits to educate about EA. Additionally, we have developed a simplified educational video on EA for dissemination on social media and recommended the inclusion of EA teaching modules in university curricula and premarital education programs.

This study had several important limitations. Its cross-sectional design captures only a snapshot in time and does not account for changes in perceptions or educational interventions that may occur throughout the pregnancy. Being a single-center study, the findings may not be generalizable across different regions or healthcare settings within Saudi Arabia or elsewhere. The reliance on self-reported data may introduce response bias, as participants might provide socially desirable answers or their recollections might be affected by their current circumstances. Additionally, excluding women who could not use the QR code technology could introduce a selection bias, potentially skewing the sample towards a more technologically savvy and possibly younger demographic. This study also does not explore the reasons behind the low rates of awareness and previous exposure to EA, which could be influenced by cultural, socioeconomic, or educational factors not accounted for in the analysis. Furthermore, the interpretation of the data is limited by the lack of qualitative insights that could provide a deeper understanding of personal beliefs and societal norms affecting attitudes toward labor analgesia.

## Conclusions

The awareness of epidural analgesia among pregnant women in Taif City is generally low, except for those working in medical professions. A correlation between previous EA exposure and C/S rates was observed. The preferred method of EA education was direct communication with obstetricians. Therefore, it is essential to enhance awareness about EA and other labor analgesia methods through comprehensive health education. This will empower women to make informed decisions regarding pain management during labor, leading to improved delivery experiences and maternal satisfaction.
